# Arthroscopic Versus Open Management of Diffuse-Type Tenosynovial Giant Cell Tumor of the Knee: A Meta-analysis of Retrospective Cohort Studies

**DOI:** 10.5435/JAAOSGlobal-D-21-00217

**Published:** 2021-12-09

**Authors:** Akhil A. Chandra, Shreya Agarwal, Ahna Donahue, Elizabeth Handorf, John A. Abraham

**Affiliations:** From the Rutgers Robert Wood Johnson Medical School, Piscataway, NJ (Mr. Chandra); Department of Surgical Oncology, Fox Chase Cancer Center, Philadelphia, PA (Mr. Chandra and Dr. Abraham); Rothman Orthopaedic Institute, Philadelphia, PA (Mr. Chandra, Ms. Agarwal, Ms. Donahue and Dr. Abraham); Drexel University School of Medicine, Philadelphia, PA (Ms. Agarwal); Biostatistics and Bioinformatics Facility, Fox Chase Cancer Center, Philadelphia, PA (Dr. Handorf) and the Sarcoma Center of Excellence, Capital Health Medical Center, Pennington, NJ (Dr. Abraham).

## Abstract

Whether arthroscopic or open surgical management for diffuse-type tenosynovial giant cell tumor (D-TGCT) of the knee is associated with a lower rate of recurrence is unknown.

**Methods:**

PubMed, Scopus, Web of Science, Cochrane, and EMBASE were searched on December 3, 2020. Retrospective studies that reported on recurrence rates for arthroscopic versus open management of D-TGCT were included. A total of 16 studies evaluating 1143 patients with D-TGCT of the knee were included (n_open_ = 551, n_arthroscopic_ = 350 patients, and n_arthroscopic/open_ = 23 patients). Random-effects meta-analyses were used to summarize and compare the reported recurrence rates, stratified by approach and overall recurrence. The meta-analysis was registered with PROSPERO.

**Results:**

The recurrence rate per year (incidence) for arthroscopic procedures was 0.11 (95% CI 0.08 to 0.16, *P* < 0.0001) and for open procedures was 0.07 (95% CI 0.04 to 0.13, *P* < 0.0001). There was a 1.56 times (95% CI 1.04 to 2.34, *P* = 0.0332) increased risk of recurrence when treating D-TGCT of the knee with an arthroscopic approach. When evaluating only the subset of studies that had data for both arthroscopic and open approaches, the incidence rate per year for arthroscopic procedures was 0.17 (95% CI 0.11 to 0.27, *P* < 0.0001) and for open procedures was 0.11 (95% CI 0.06 to 0.19, *P* < 0.0001). The rate of overall complications was 0.04 (95% CI 0.01 to 0.08, *P* < 0.0001).

**Conclusion:**

Arthroscopic surgical management of D-TGCT of the knee in our study resulted in a 1.56 times risk of recurrence as compared with the open approach. The percent of overall complications was minimal.

Tenosynovial giant cell tumor (TGCT), formerly known as pigmented villonodular synovitis, is a rare benign synovial neoplasm, which leads to overgrowth of the synovium lining joints, eventually resulting in cartilage loss and bone erosion.^1-3^ The estimated annual incidence is 1.8 per million, with presenting symptoms typically arising in the third to fourth decade of life.^2,4^

TGCT may present in a localized (L-TGCT) or diffuse (D-TGCT) form.^4^ L-TGCT is a well-defined solitary tumor arising from the synovium, whereas D-TGCT permeates the entire synovium, sometimes extending into extra-articular spaces; thus, D-TGCT generally confers a much poorer prognosis.^2,3,5^ Studies have shown that D-TGCT has been associated with more technically challenging procedures, with recurrence rates reaching as high as ∼50%.^4-6^ The knee is the most affected joint (>70% of the cases); patients commonly present with pain, effusion, and joint dysfunction.^4^

MRI is the benchmark for evaluating the disease, demonstrating a diffusely thickened synovium with villous finger-like projections. The synovium may show areas of hypointensity on T1-weighted and T2-weighted images from hemosiderin deposition, which gives the tumor its characteristic rust-colored appearance on gross examination.^2,7,8^ On histologic evaluation, D-TGCT demonstrates multinucleated osteoclast-like giant cells, foamy macrophages, and hemosiderin-laden macrophages.

Recent literature has identified chromosome 1p13 translocation in most TGCT cases.^9,10^ The resulting translocation has been linked to overexpression of Colony Stimulating Factor (CSF)-1, a gene that mediates the proliferation and recruitment of macrophages (a predominant cell-type in TGCT). Overexpression of CSF-1 has been purported to recruit CSF1R-expressing macrophages, thus creating a “landscape” effect of tumor proliferation from nonneoplastic cells. Therefore, the recent finding of CSF-1 as the driver mutation for this condition has led to the development of medical therapies as alternatives to surgical excision.^3,9,11^

The standard of care for resectable tumors has historically been surgical excision. Surgery is generally curative and leads to low rates of recurrence for L-TGCT.^12^ However, studies on the effectiveness of surgery for D-TGCT have been relatively limited, and consensus regarding whether arthroscopic or open surgical management produces the best results has not yet been reached. Current literature provides arguments for both modalities, leaving the question unanswered.

Given the recently available option of pexidartinib for unresectable or poorly surgically controlled tumors, it is critical to understand the outcomes and limitations of surgical excision. By understanding which method of surgical excision forms the benchmark for comparison with medical therapy, clinicians may better decide whether medical or surgical therapy is most effective for the treatment of D-TGCT.^13^ The purpose of this meta-analysis was to determine, based on current available and assessable literature, whether arthroscopic or open excision of D-TGCT specifically of the knee results in a lower recurrence rate.

## Methods

The PRISMA 2009 guidelines were followed for this study design and reporting.

### Protocol and Registration

Methods of the search strategy and inclusion criteria were specified in advance and documented in a protocol published in PROSPERO's International Prospective Register of Systematic Reviews (https://www.crd.york.ac.uk/prospero/display_record.php?ID=CRD42021231130).

### Information Sources and Search Strategy

On December 3, 2020, we searched PubMed, Scopus, Web of Science, Cochrane, and EMBASE databases for search terms ([pigmented villonodular synovitis] OR [TGCT)] AND [Knee Joint] AND ([Surgery] OR [Arthroscopy]). Whole names and acronyms were used when conducting this literature search. There were no date limits. All studies consist of peer-reviewed, English-language articles. All searches were exported into an EndNote library for data extraction.

### Eligibility Criteria

We included studies that reported on recurrence rates for arthroscopic versus open management of D-TGCT. If D-TGCT and the associated recurrence rates for the surgical approach were not mentioned in the abstract or title, the study was not included. Studies without abstracts or studies which focused on anatomical sites that did not include the knee were excluded. Case reports, letters to the editor, conference proceedings, and literature reviews were excluded. We did not exclude studies based on sample size.

### Study Selection

**Figure 1:**
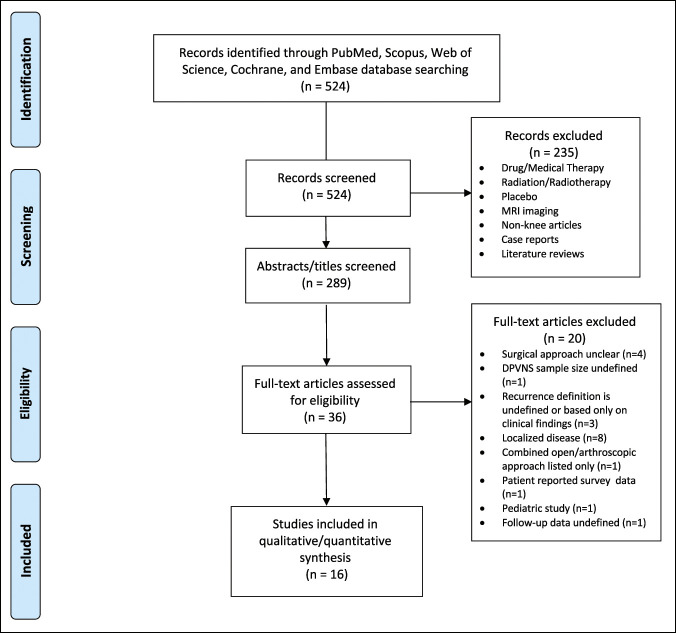
CONSORT diagram showing literature search.

After removing duplicates, three authors (A.A.C., S.A., and A.D.) partitioned and read both the title and abstract of the remaining 289 articles to determine whether the article met inclusion criteria. The authors did an initial screening through abstracts to ensure that proper data were available before reviewing full-text articles for data collection. After preliminary review of abstracts, 36 full-text articles were selected for review and data extraction (Figure 1).

### Data Extraction

Three authors (A.A.C., S.A., and A.D.) reviewed the full-text articles for the 36 selected studies. The lead author (A.A.C.) and corresponding author (J.A.A.) then re-reviewed the selected studies after data collection was completed to ensure that no data were missing or lost and narrowed the final study list to 16 articles. From the final 16 articles, the following data elements were extracted: publication lead author, publication year, study design, study start/end year, number of patients by diagnosis of D-TGCT and surgical approach, mean/median age, recurrence rates by the surgical approach, mean/median follow-up time, minimum/maximum follow-up time, and overall complication rate.

### Statistical Analysis

We used random-effects meta-analyses to summarize the reported recurrence rates, both stratified by approach and overall. The reported recurrence rates were used to calculate log-transformed incidence rates per year where the time at risk was calculated using the reported follow-up time (study time at risk = study population*mean years of follow-up). For studies with both arthroscopic and open cases reported, we also calculated the incidence rate ratio for recurrence in arthroscopic versus open approaches. Complication rates were estimated as proportions based on models for proportions, using random-effects meta-analyses with a logit transformation. For each outcome, a study was included provided that it reported sufficient information to analyze that particular outcome. Analyses were conducted in R version-3.6 using the metafor and meta packages.^14,15^

## Results

After procuring 524 articles on first screen, 16 studies met inclusion criteria, whereas 235 abstracts and 20 full-text articles were excluded (Figure 1). The 16 studies included in the final analysis were retrospective cohort studies that evaluated arthroscopic and/or open surgical management of D-TGCT and listed an associated recurrence rate (Table 1). Four studies evaluated open approach, six evaluated arthroscopic approach, five evaluated both arthroscopic and open approaches, and one did not specify any approach but was included because of author preference.

**Table 1:** Characteristics of the Studies Included and Results Summary

Author	Year	Study Design	Study Start Year	Study End Year	N Diffuse + Localized	N Diffuse Only	N Open	N Arthroscopic	N Open/Arthroscopic Combined	Mean Age (Min-Max, years)	Overall Recurrence Rate	Recurrence Rate Open	Recurrence Rate Arthroscopic	Recurrence Rate Open/Arthroscopic Combined	Mean Follow-up Time (Min-Max, mo)	Median Follow-up Time (Min-Max, mo)	Overall Complication Rate
Akinci et al	2011	Retrospective	1996	2009	19	15	15			42.8 (15-62)		0.26			80.2 (15-156)		0
Auregan et al	2013	Retrospective	1998	2011	23	7		7		41			0.29		84.0		0.04
Capellen et al	2018	Retrospective	1996	2014	105	36	36			42 (12-82)		0.22				71.0 (13-238)	0.06
Chin et al	2002	Retrospective	Not Listed	Not Listed	40	40	5			35.8	0.00				60.0		
Colman et al	2013	Retrospective	1993	2011	103	48	11	26	11	Open—24 Arthro- 32 Combined—34	0.50	0.64	0.62	0.09		40.0 (3-187)	0.13
De Ponti et al	2003	Retrospective	1990	1999	19	15		15		59 (37-83)			0.50		60.0 (12-128)		0
Jain et al	2013	Retrospective	1987	2012	40	29		29		44 (21-76)			0.41		84.0 (24-120)		0
Ma et al	2013	Retrospective	2000	2010	75	44		44		46 (15-80)			0.25		41.0		0
Mastboom et al	2019	Retrospective	1990	2017	559	559	358	96		35 (26-48)		0.37	0.43			54.0 (27-97)	0.12
Ogilvie et al	1992	Retrospective	1979	1989	25	20		20		38 (17-80)			0.30		54.0 (24-120)		0
Patel et al	2017	Retrospective	2002	2015	214	114	83	12	4	39	0.48	0.45	0.83		25.0 (2-168)		0.10
Sharma, Cheng et al	2009	Retrospective	1991	2008	49	37	16	13	8	35.2 (10-73)	0.46	0.33	0.92		74.4 (12-156)		
Sharma, Rana et al	2007	Retrospective	1950	2000	16	13	13			33 (16-58)		0.19			72.0 (12-168)		0
van der Heijden et al	2014	Retrospective	1980	2001	30	30	14	16		34 (6-73)		0.29	0.94		64.0 (24-393)		0.07
Verspoor et al	2014	Retrospective	1985	2011	107	64				Male—32.1 Female—35.7	0.44				156.0 (13-584)		0.30
Zvijac et al	1999	Retrospective	1987	1997	14	12		12		34.6 (19-64)			0.14		42.0 (8-83)		0

A total of 16 studies evaluating 1143 patients with D-TGCT of the knee were included in the final analysis. Of the 1143 patients, 551 were identified as undergoing open approach, 350 as arthroscopic approach, and 23 as a combined arthroscopic and open approach. The mean follow-up time for our entire cohort was 58.3 months (range 2 to 584 months), whereas the mean follow-up time for our arthroscopic cohort and open cohort was 55.5 months (range 2 to 393 months) and 58.5 months (range 3 to 393 months), respectively. The mean age of our cohort was 38.8 years (range 6.0 to 83.0). Recurrence rates for the open approach ranged from 0.19 to 0.64, recurrence rates for the arthroscopic approach ranged from 0.14 to 0.94, and recurrence rates for the combined approach ranged from 0.44 to 0.50 (Table 1). Only three studies with a collective sample size of 23 patients reported data on combined open and arthroscopic approach; thus, these patients were not included in the meta-analysis to determine recurrence rates^16-18^ (Table 1).

Owing to the low-medium quality of the retrospective reviews and heavy bias across studies, the studies included in this meta-analysis were not assessed for heterogeneity using a formal scale.

### Primary Outcomes—Recurrence Rates by Approach

#### Arthroscopic

**Figure 2:**
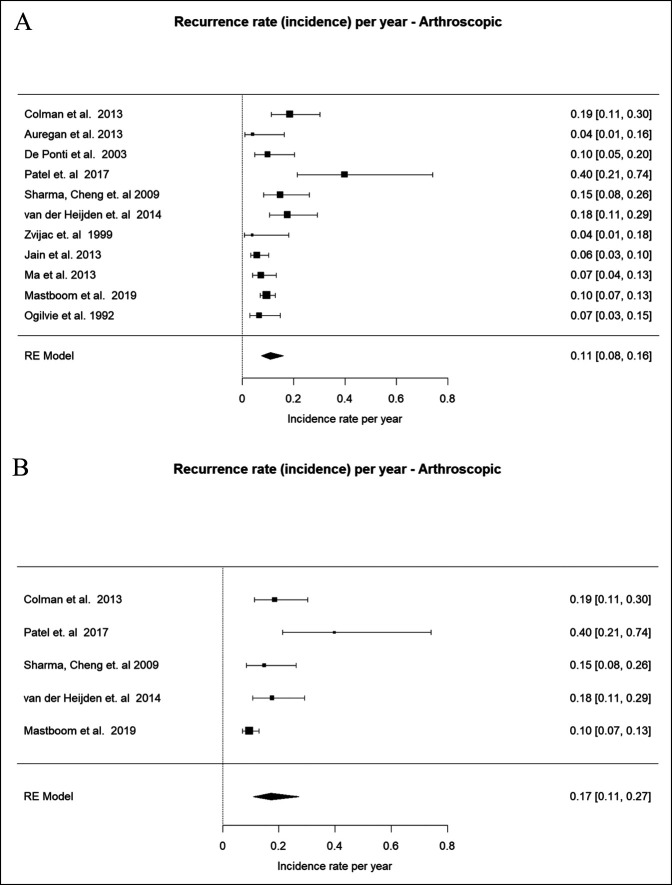
Forest Plot showing recurrence rate for arthroscopic approach (**A**) and its subset (**B**).

The incidence rate (or recurrence rate) per year for arthroscopic procedures (n = 11) was 0.11 (95% CI 0.08 to 0.16, *P* < 0.0001)^16, 19, 20, 17, 18, 21,22,23,24,25,26^ (Figure 2A). When evaluating only the subset that had data for both arthroscopic and open approaches (n = 5), the incidence rate per year was 0.17 (95% CI 0.11 to 0.27, *P* < 0.0001)^16-18, 21, 25^ (Figure 2B).

#### Open

**Figure 3:**
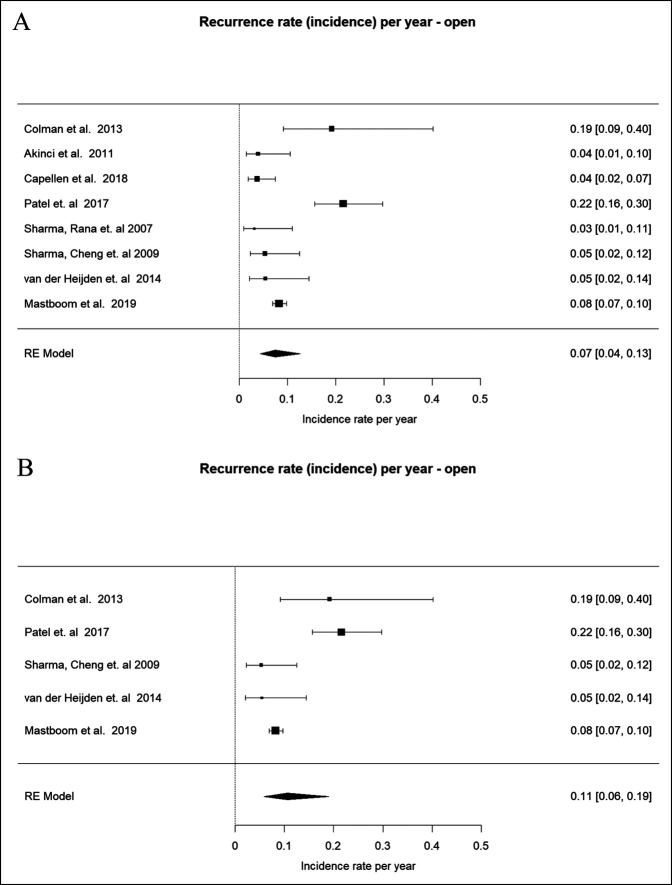
Forest Plot showing recurrence rate for open approach (**A**) and its subset (**B**).

The incidence rate (or recurrence rate) per year for open procedures (n = 8) was 0.07 (95% CI 0.04 to 0.13, *P* = <0.0001)^16, 27, 28, 17, 29, 18, 21, 25^ (Figure 3A). When evaluating only the subset that had data for both arthroscopic and open approaches (n = 5), the incidence rate per year was 0.11 (95% CI 0.06 to 0.19, *P* < 0.0001) ^16-18, 21, 25^ (Figure 3B).

#### Arthroscopic Versus Open

**Figure 4:**
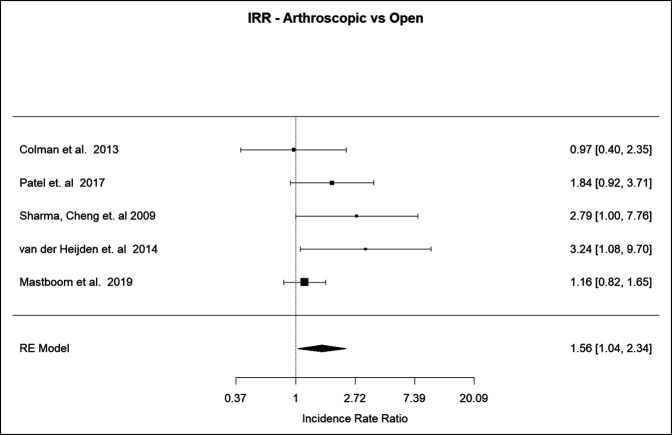
Graph showing the recurrence rate for arthroscopic versus open approach.

The incidence rate ratio was 1.56 (95% CI 1.04 to 2.34, *P* = 0.0332), denoting that there was a 1.56 times increased risk of recurrence when treating D-TGCT of the knee with the arthroscopic approach^16-18,21,25^ (Figure 4).

#### Overall Recurrence Rate

**Figure 5:**
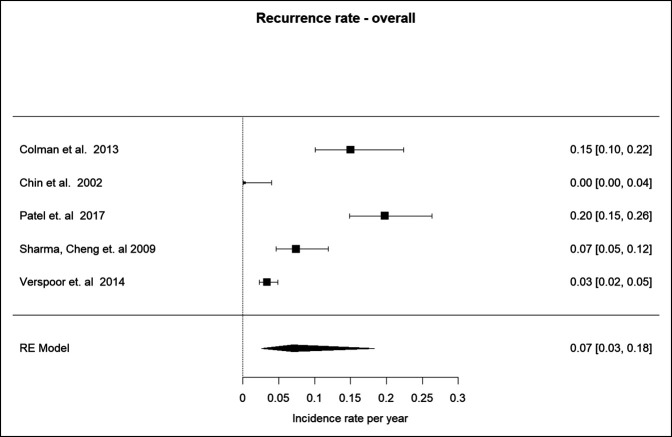
Graph showing the overall recurrence rate.

The overall recurrence rate for studies included both approaches and reported that a combined rate (n = 5) was 0.07 (95% CI 0.03 to 0.18, *P* < 0.0001)^16-18,21,25^ (Figure 5).

#### Secondary Outcome—Complication Rates

**Figure 6:**
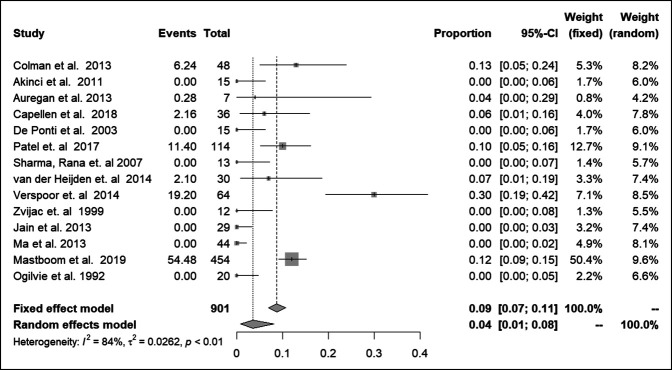
Graph showing complication rates for overall cohort.

The proportion of overall complications in the entire cohort combining both arthroscopic and open approaches (n = 14), was 0.04 (95% CI 0.01 to 0.08, *P* < 0.0001)^16, 27, 19, 28, 20, 17, 29, 21, 30, 22,23,24,25,26^ (Figure 6).

## Discussion

D-TGCT of the knee is a rare and aggressive neoplastic process that has been associated with overexpression of CSF-1.^31^ This condition purports a poor prognosis, with recurrence rates after surgical excision as high as 50%.^4-6^ Although previous studies have commented on arthroscopic versus open resection for the surgical management of D-TGCT, consensus regarding which approach is most effective has not been reached. Owing to the recent advent of novel CSF-1 receptor inhibitors for the medical management of D-TGCT, a more comprehensive understanding of the benefits and disadvantages of surgical excision has become necessary.

To the best of our knowledge, this meta-analysis is the largest study to report on arthroscopic versus open surgical management of D-TGCT of the knee. Based on our analysis, arthroscopic approaches result in a 1.56 times greater risk of recurrence (recurrence rate per year [incidence] for arthroscopic procedures was 0.11 [95% CI 0.08 to 0.16, *P* < 0.0001] and open procedures was 0.07 [95% CI 0.04 to 0.13, *P* < 0.0001], absolute risk reduction = approximately 0.04), as compared with open approaches when treating D-TGCT of the knee. The rate of overall complications was determined to be approximately 4.0%. Our findings are in accordance with previous reports which have suggested that an open approach results in lower rates of recurrence when compared with an arthroscopic approach for the treatment of D-TGCT of the knee.^17,18,21,25^ However, although these reports have commented on the benefits of an open approach, because of their retrospective nature and limited sample sizes, clear conclusions regarding recurrence rates were unclear. Our study is the first to report on an aggregated patient population using a random-effects meta-analysis model, thus providing a higher level of evidence to support the use of an open approach when treating D-TGCT of the knee.

The arthroscopic recurrence rates found in our study (11 to 17%) are similar to the rates previously reported in the literature. Although the retrospective, multicenter study by Mastboom et al.^25^ did not note a difference in first local recurrence based on a surgical technique in therapy-naïve patients with D-TGCT of the knee (n = 471, *P* = 0.11), the increased risk of an arthroscopic approach has been reported by other groups. Patel et al.^17^ and Chin et al.^32^ both commented on poorer outcomes from arthroscopic management of D-TGCT. Patel et al. noted a statistically significant increase in recurrence rates when performing subgroup analysis comparing arthroscopic versus open approach (83.3% versus 44.8%, RR = 1.86, 95% CI 1.32 to 2.62, *P* = 0.0004). Chin et al. determined that nearly 90% of their group's arthroscopic cohort had worse outcomes at mean 3.63-year follow-up and advised against the use of arthroscopic approach, regardless of its perceived decreased postoperative morbidity.

There is a paucity of literature on combined arthroscopic and open approach for treatment of D-TGCT of the knee. Mollon et al.^6^ noted a lower rate of recurrence in a cohort treated with combined arthroscopic and open synovectomy as compared with arthroscopic alone (OR=0.19, 95% CI=0.06 to 0.58; *P* = 0.003). Colman et al^16^ noted that in a cohort of 48 patients with D-TGCT of the knee treated with arthroscopic (n = 26), open (n = 11), or combined approach (n = 11), the recurrence rates were lower in the combined group compared with the arthroscopic or open groups (9% versus 62% versus 64%, respectively). None of the patients (n = 4) with D-TGCT of the knee treated with the combined approach in the cohort of Patel et al^17^ developed recurrence. Sharma et al. also studied eight patients with D-TGCT of the knee that underwent the combined approach but did not draw strong conclusions because they noted that recurrence was frequent, regardless of the surgical approach.^18^ A systematic review by Healey et al^33^ concluded that interpretation of studies presenting data on a combined approach is limited by study design limitations and lack of controlling for differences in surgeon expertise and patient clinical characteristics, among other confounders.

When evaluating complication rates after surgery for D-TGCT of the knee in our study, overall complication rates (which included hemarthrosis, DVT, dehiscence, arthrofibrosis, infection, foot drop, necrosis, and other complications) were ∼4.0%. We noted that some articles did not report pain, joint stiffness, or scarring as outcomes that have been frequently cited to be a reason for the use of an arthroscopic approach in patients.^20^

Previous studies have commented on the potential etiologies for the increased recurrence rates in arthroscopic surgery, with some suggesting that it is due to an incomplete resection of the neoplastic tissue.^18,20^ West et al. reported that the overexpression of CSF-1 in a minority of cells is the causative factor of D-TGCT, a finding that gives biologic plausibility to the suggestion that a suboptimal resection, such as with arthroscopic surgery, may leave neoplastic driver cells behind and thereby increase risk of recurrence.^9,11^ De Ponti et al^20^ reported that the steep learning curve for being able to execute a radical resection of a D-TGCT lesion arthroscopically may account for the variation in recurrence rates. It is also possible that arthroscopically shaving the synovium, which is generally the method of resection rather than actual removal of neoplastic tissue as in open surgery, may increase the level of CSF-1 in the joint by forcing a “mechanical paracrine” effect: Shaving neoplastic cells with high levels of intracellular CSF-1 may cause cell destruction and increased release of CSF-1 into the joint. Although the exact etiology for increased rates of recurrence in arthroscopic surgery may be unclear, we think that achieving adequate gross surgical excision, which is better facilitated by an open approach, is a contributing factor in improved local control using an open method.^34,35^ Our findings are in line with a previous systematic review by van der Heijden et al,^36^ which proposed open complete synovectomy for the treatment of D-TGCT, although that study also suggested the addition of external beam radiation.

With the emergence and implementation of CSF-1 receptor inhibitors, such as pexidartinib, it has become essential to understand the efficacy of arthroscopic versus open approach for managing D-TGCT so that comparisons may eventually be drawn between surgical and medical therapy.^13,31^ This is critically important because the medication is approved for “unresectable” disease, patients not appropriate for surgery. To know who best fits that indication, it is necessary to know what the limitations of surgical resection are for this disease. In our study, we report that the incidence of recurrences per year for arthroscopic procedures (0.11 to 0.17) was greater than that of open procedures (0.07 to 0.11). Because additional research is published on the long-term results of CSF-1 receptor inhibitors, future goals should focus on comparing whether a medical approach may lead to a lower rate of recurrence when compared with an open surgical approach or whether there may be a role for a combined medical/surgical approach, such as administration of the drug in a neoadjuvant or adjuvant setting in combination with surgery.

There are several limitations to this study. First, we noted that the recurrence rates of both arthroscopic and open approaches were higher when we evaluated only studies that reported both approaches in their sample population. Thus, the increase in recurrence rates may indicate that there was a clinical difference in the patient populations across the studies. Second, heterogeneity of patient cohorts across studies was not accounted for because of the low-medium quality of the few retrospective reviews that have been published on this topic and heavy bias across studies. Third, the decision making which would explain why each patient received a specific approach is not mentioned in studies. Fourth, we could not control for confounding factors that would influence the rates of recurrence, such as surgeon expertise and hospital volume. Fifth, the results of this study were presented as incidence rates per year; thus, the overall rates of recurrence when accounting for a longer follow-up period may be even greater. Finally, we did not account for adjuvant therapies in this study, which would affect recurrence rates in this patient population; this was done so that the surgical approach alone could be compared without risk of including adjuvant therapy as a confounding factor. Future studies should use randomized trials to account for these limitations so that they may decrease the risk of bias when evaluating the best approach to manage D-TGCT of the knee.

## Conclusions

Arthroscopic surgical management of D-TGCT of the knee in our study resulted in a 1.56 times risk of recurrence as compared with the open approach. The percent of overall complications (4.0%) was minimal. Thus, open surgical excision may offer benefit over arthroscopic excision for the treatment of D-TGCT of the knee. Future studies should use randomized trials to evaluate the effect of surgical approach on rates of recurrence and complications when treating D-TGCT of the knee and compare surgical approach with medical management. Clinicians should factor patient preference and other treatment factors through a model of “shared decision making” to determine the optimal treatment plan for each patient.
